# Postoperative complications of combined phacoemulsification and pars plana vitrectomy in diabetic retinopathy patients

**DOI:** 10.3389/fmed.2022.978346

**Published:** 2022-09-30

**Authors:** Assaf Gershoni, Edward Barayev, Doha Jbara, Amir Hadayer, Ruth Axer-Siegel, Assaf Dotan, Orly Gal-Or, Raimo Tuuminen, Rita Ehrlich

**Affiliations:** ^1^Department of Ophthalmology, Rabin Medical Center, Petah Tikva, Israel; ^2^Sackler Faculty of Medicine, Tel Aviv University, Tel Aviv, Israel; ^3^Helsinki Retina Research Group, University of Helsinki, Helsinki, Finland; ^4^Department of Ophthalmology, Kymenlaakso Central Hospital, Kotka, Finland

**Keywords:** non-proliferative diabetic retinopathy (NPDR), cystoid macular edema (CME), phacoemulsification (phaco), proliferative diabetic retinopathy (PDR), vitrectomy

## Abstract

**Purpose:**

To compare intra- and postoperative complications in combined phacoemulsification and pars plana vitrectomy surgeries performed in patients with non-proliferative diabetic retinopathy (NPDR) vs. proliferative diabetic retinopathy (PDR).

**Methods:**

Retrospective, case series of patients with diabetic retinopathy who underwent combined phacovitrectomy surgery between 2008 and 2017. We compared intraoperative complications including posterior capsular rupture and retinal tear, and postoperative complications including corneal edema, macular edema (ME), epiretinal membrane (ERM), neovascular glaucoma and persistent inflammation.

**Results:**

A total of 104 eyes of 104 patients were included in this study. Twenty-four eyes (23.1%) were categorized as NPDR and 80 eyes (76.9%) as PDR. The most common indications for surgery in the NPDR group were ERM (67%) and rhegmatogenous retinal detachment (12.5%), while in the PDR group, indications were vitreous hemorrhage (56%) and tractional retinal detachment (19%). The most common intraoperative complication was retinal tear (8% in NPDR and 19% in PDR, *p* = 0.195) and postoperative complication was ME (29% in NPDR and 26% in PDR, *p* = 0.778). There were no statistically significant differences in intra- and postoperative complication rates between the NPDR and PDR groups, even after adjusting for confounders; patient age at surgery and indication for surgery.

**Conclusion:**

After combined phacovitrectomy in NPDR and PDR patients, new-onset ME was found in about a quarter of eyes in both groups. Intraoperative anti-VEGF or steroid administration, and intense postoperative anti-inflammatory medication and follow-up should be regarded after phacovitrectomy regardless of the DR level.

## Introduction

Diabetic retinopathy (DR) is the most common microvascular complication of diabetes, and is one of the leading causes of blindness worldwide (1). Both the prevalence and severity of DR correlate strongly with the duration of diabetes and poor glycemic control (2).

The Early Treatment Diabetic Retinopathy Study (ETDRS) established uniform criteria for classifying DR, based on the severity of the disease (3). Non-proliferative DR (NPDR) is defined by the presence of hemorrhages, microaneurysms and hard exudates and is categorized as mild, moderate, or severe. Proliferative DR (PDR) is distinguished from NPDR by the presence of neovascularization (NV) on the iris, optic disc, or the retina. NV is typically accompanied by proliferation of fibrovascular tissue and glial cells with contractile properties that may lead to tractional retinal detachment (TRD). Vitreous hemorrhages, TRDs, and diabeticmacular edema (DME) represent some of the complications of DR, and constitute the leading causes of vision deterioration in DR (4). In such cases, surgical intervention may be warranted.

Since the rate of cataract formation is three times more prevalent in patients with diabetes, vitreoretinal diseases frequently coexist with cataract among these patients (5). Furthermore, there is a high frequency of cataract formation following pars plana vitrectomy (6). Thus, combined surgery of phacovitrectomy may provide an adequate surgical treatment for such patients.

The advantages of combined surgery include better visualization during the operation and relinquishing the need for additional cataract surgery in the future (7, 8). Another argument favoring combined surgery is that future cataract surgery might be more challenging in vitrectomized eyes due to an extremely deep anterior chamber and zonular dehiscence (7). Combined surgery has its disadvantages as well, as it is a more challenging operation with longer duration, and therefore prone to intra- and postoperative complications (9, 10).

Currently, limited information is available regarding the outcomes and complications of combined phacovitrectomy in patients suffering from DR. In this study, we aimed to compare the intra- and postoperative complications of combined phacovitrectomy procedures between the patients with NPDR and PDR.

## Materials and methods

This is a retrospective comparative observational study. The study included patients who were operated in a single tertiary medical center between January 2008 and August 2017. The study was approved by the Rabin Medical Center Institutional Research Ethics Board and adhered to the tenets of the Declaration of Helsinki. Informed consent was waived by the Rabin Medical Center Institutional Review Board due to the retrospective nature of the study.

### Study cohort

The study group consisted of patients with DR and cataract who underwent combined pars plana vitrectomy, phacoemulsification and subsequent implantation of a foldable IOL. All combined procedures were indicated for visualization purposes during the surgery, and in order to achieve an easier and better peripheral vitrectomy.

Patients were divided into two groups depending on disease classification—NPDR or PDR. The NPDR group included patients with any sign of diabetic retinopathy without the presence of neovascularization and underwent combined surgery for other retinal coexisting pathologies including ERM, RD, etc. (Table 1) (17).

**Table 1:** Demographic characteristics.

	**NPDR** **(*n* = 24)**	**PDR** **(*n* = 80)**	***P*-value**
Mean age (years)	71.4 ± 7.0	65.0 ± 10.1	**0.006**
Male/female	11/13	50/30	0.146
Hypertension	14 (58)	39 (49)	0.684
Coronary vascular disease	7 (29)	26 (32)	0.646
**Indication for surgery**			
Vitreous hemorrhage	1 (4)	45 (56)	
Epiretinal membrane	16 (67)	11 (14)	
TRD	0	15 (19)	**<0.001**
RRD	3 (12.5)	3 (4)	
Vitreomacular traction	3 (12.5)	1 (1)	
Other	1 (4)	5 (6)	
**Intraoperative laser/anti-VEGF**			
PRP	9 (38)	53 (66)	**0.012**
Anti-VEGF injection	6 (25)	29 (36)	0.306

Data is given as mean ± SD for continuous variables and absolute numbers with proportions for categorical variables. For two-group comparisons, parametric variable (age) was analyzed with the Student's *t*-test. Categorical data was analyzed with the Pearson's chi-square test or the Fisher's exact test when values in any of the cells of a contingency table were below five. NPDR, non-proliferative diabetic retinopathy; PDR, proliferative diabetic retinopathy; PRP, pan-retinal photocoagulation; RRD, rhegmatogenous retinal detachment; TRD, tractional retinal detachment; VEGF, vascular endothelial growth factor. *p*-values < 0.05 were considered statistically significant.

Included were patients 18 years or older, diagnosed with either NPDR or PDR, who had undergone combined pars plana vitrectomy and phacoemulsification, had a complete medical record including general medical and ocular history, slit-lamp examination, intraocular pressure (IOP), best-corrected visual acuity (BCVA), and were followed for 6 months after the surgery. Exclusion criteria included previously vitrectomized eyes or previous ocular trauma, any preoperative neovascularization of the iris/iridocorneal angle and patients who were lost to follow-up during the first 6 months after surgery.

### Clinical evaluation

The medical files of the patients were reviewed for demographics (age, gender, ocular history and coexisting systemic hypertension and ischemic heart disease), BCVA and IOP prior to surgery and during the follow-up visits. All patients underwent a comprehensive ophthalmic examination and optical coherence tomography (OCT) analysis by the retina specialist referring to surgery.

Perioperative data included indications for vitreoretinal surgery, use of intraoperative laser/anti-VEGF and any intraoperative complications such as posterior capsular rupture or retinal tears. Additionally, postoperative complications such as corneal edema, macular edema (ME), epiretinal membrane (ERM), neovascular glaucoma (NVG), persistent inflammation, persistent ocular hypertension, posterior capsular opacification (PCO), posterior synechiae, retinal detachment (RD) and vitreous hemorrhage (VH) were recorded.

All patients had their DME in a quiescent state before being referred to surgery. Macular edema was defined as the existence of intraretinal fluid on OCT, and an increase of 60 μm in central macular thickness (CMT; defined as the mean macular thickness in the central 1,000 μm area) (18).

### Surgical technique

Surgery was performed by one of three experienced retinal surgeons using the Stellaris Vitreoretinal Surgical System (Bausch & Lomb, Rochester, NY) with wide-angle viewing, using 25-gauge instruments. In all surgeries, phacoemulsification preceded the vitreoretinal procedure. When IOL calculations were not attainable in cases of retinal detachments or vitreous hemorrhages, axial length was calculated utilizing A-scan ultrasound while comparing the results to full optical biometry of the contralateral eye. After phacoemulsification and IOL implantation through the main corneal incision, viscoelastic material was completely aspirated. In order to avoid anterior chamber collapse during vitrectomy procedures, a prophylactic 10-0 nylon suture was placed on the clear corneal wound when needed. Following the cataract surgery, a standard 3-port 25-gauge pars plana vitrectomy was performed. When the indication for surgery was ERM, it was peeled off with the internal limiting membrane (ILM). No routine peel of the ILM was performed for any other indication. At the end of surgery, fluid air exchange and gas injections were performed if retinal breaks were found. Endolaser was added in PDR cases if panretinal photocoagulation was not complete, and on areas of capillary non-perfusion in cases of severe NPDR, according to the surgeon's decision (13, 19, 20). Patients with PDR and preproliferative NPDR received anti-VEGF injections at the end of surgery at the surgeon's discretion. All patients received topical antibiotics q.i.d. for 2 weeks, topical steroids 4–8 times per day postoperatively with gradual tapering down, and topical non-steroidal anti-inflammatory drugs (NSAIDs) t.i.d. for 3 months postoperatively. The patients did not receive topical steroids or NSAIDs preoperatively. All patients underwent macular OCT 1 month postoperatively unless non-resolving VH prevented visualization.

Primary outcome measures of the study were intra- and postoperative complications.

### Statistical analysis

For two-group comparisons, parametric variables were analyzed with the Student's *t*-test and non-parametric data with the Mann-Whitney U test. Categorical data was analyzed with the Pearson's chi-square test or the Fisher's exact test when values in any of the cells of a contingency table were below five. Multivariate analyses of variance (MANOVAs; with appropriate mixed-model error terms) was conducted to evaluate the effect of DR type and surgery indication on any intraoperative or postoperative complications, accounting for age. Data is presented as absolute numbers and proportions for categorical variables, and as means and standard deviation for continuous variables. SPSS v.27.0 (IBM Corp., Armonk, NY) was used to analyze the data. *P*-values < 0.05 were considered statistically significant.

## Results

### Baseline variables

One hundred and four eyes of 104 patients were included in the study. Twenty-four eyes (23.1%) had NPDR and 80 eyes (76.9%) had PDR.

Mean age at time of surgery was significantly higher in the NPDR than in the PDR group (71.4 ± 7.0 vs. 64.9 ± 10.1, *p* = 0.006). The patients' demographics are presented in Table 1. Six of the patients were previously treated for DME with anti-VEGF injections, and four of them were treated in the preoperative period (6 months). All patients responded to treatment before being referred to surgery.

The most common indications for surgery in the NPDR group were ERM (67%), rhegmatogenous retinal detachment (RRD, 12.5%) and vitreomacular traction (VMT, 12.5%). In the PDR group VH (56%), TRD (19%), and ERM (14%) were the most common indications (*P* < 0.001, Table 1). Intraoperative PRP was administered in 38% of the eyes (*n* = 9) in the NPDR group and in 66% of the eyes (*n* = 53) in the PDR group for completion of PRP (Table 1). All patients who received intraoperative PRP in the NPDR group had the severe form of the disease.

### Clinical outcomes of phacovitrectomy in NPDR and PDR eyes

Transient postoperative IOP elevation (>21 mmHg) was observed in 29 eyes (27.9%) of the entire cohort, 22 of which were in the PDR group. No statistically significant differences in IOP between the groups were found during the follow-up (Table 2). Preoperative BCVA in the PDR group was significantly worse than in the NPDR group (1.26 ± 0.73 vs. 0.80 ± 0.59 LogMAR units, *p* = 0.005, Table 2), whereas postoperative BCVA was comparable between the study groups (Table 2).

**Table 2:** Visual acuity and intraocular pressure of NPDR and PDR patients after combined phacoemulsification and 25G vitrectomy.

	**NPDR** **(*N* = 24)**	**PDR** **(*N* = 80)**	***P*-value**
**BCVA (LogMAR)**			
Baseline	0.80 ± 0.59	1.26 ± 0.73	**0.005**
1-month	0.84 ± 0.56	0.94 ± 0.62	0.456
3-month	0.78 ± 0.55	1.09 ± 1.51	0.379
6-month	0.78 ± 0.62	0.86 ± 0.72	0.618
**IOP (mmHg)**			
Baseline	14.6 ± 2.2	14.9 ± 3.1	0.608
1-day	18.7 ± 9.1	18.4 ± 10.4	0.934
1-month	14.4 ± 3.7	14.4 ± 4.2	0.995
3-month	15.5 ± 4.5	14.2 ± 5.7	0.378
6-month	14.8 ± 4.2	14.7 ± 6.1	0.937

Data is given as mean ± SD. For two-group comparisons, continuous and normally distributed data (IOP) was analyzed with the Student's *t*-test, and non-parametric data (BCVA) with the Mann-Whitney U test. BCVA, best-corrected visual acuity; IOP, intraocular pressure; LogMAR, logarithm of the minimum angle of resolution; NPDR, non-proliferative diabetic retinopathy; PDR, proliferative diabetic retinopathy.

*p*-values < 0.05 were considered statistically significant.

### Complications

The most common intraoperative complications were retinal tears observed in 8% of the NPDR and in 19% of the PDR eyes (*p* = 0.195, Table 3) and posterior capsular rupture in 5% of the PDR eyes (Table 3). No posterior capsule rupture was observed in the NPDR group. As for postoperative complications, ME was the most common complication in both groups (29.2 and 26.2%, respectively, *p* = 0.778, Table 4), followed by the formation of ERM (16.7 and 17.5%, respectively, *p* = 1.000). Out of the 28 patients who had postoperative macular edema, 10 (35.7%) had OCT characteristics compatible with some degree of DME as well, 4 of which were in the NPDR group and 6 in the PDR group. Six patients (5.8%) had some degree of preoperative non-central-involving DME. The groups did not differ in overall rates of any postoperative complications (63% in NPDR vs. 64% in PDR, *p* = 0.911, Table 4). Corneal edema, persistent inflammation and neovascular glaucoma were observed only in the PDR eyes, although these differences were not statistically significant.

**Table 3:** Intraoperative complications of combined phaco-vitrectomy in patients with NPDR and PDR.

	**NPDR (*N* = 24)**	**PDR** **(*N* = 80)**	***P*-value**
Any intraoperative complication	2 (8)	19 (24)	0.147
Posterior capsule tear	–	4 (5)	0.570
Retinal tear	2 (8)	15 (19)	0.195

Data is given as absolute numbers with proportions. For two-group comparisons, categorical data was analyzed with the Pearson's chi-square test or the Fisher's exact test when values in any of the cells of a contingency table were below five. NPDR, non-proliferative diabetic retinopathy; PDR, proliferative diabetic retinopathy.

**Table 4:** Postoperative complications of combined phaco-vitrectomy in patients with NPDR and PDR.

	**NPDR (*N* = 24)**	**PDR** **(*N* = 80)**	***P*-value**
**Any postoperative complication**	15 (63)	51 (64)	0.911
Corneal edema	–	3 (4)	0.430
Postoperative macular edema	7 (29)	21 (26)	0.778
Epiretinal membrane	4 (17)	14 (18)	1.000
Neovascular glaucoma	–	3 (4)	0.430
Persistent inflammation	–	2 (3)	1.000
Persistent ocular hypertension	1 (4)	11 (14)	0.156
Posterior capsule opacification	2 (8)	7 (9)	0.842
Posterior synechiae	1 (4)	4 (5)	0.643
Retinal detachment	1 (4)	7 (9)	0.676
Vitreous hemorrhage	1 (4)	8 (10)	0.449

Data is given as absolute numbers with proportions. For two-group comparisons, categorical data was analyzed with the Pearson's chi-square test or the Fisher's exact test when values in any of the cells of a contingency table were below five. NPDR, non-proliferative diabetic retinopathy; PDR, proliferative diabetic retinopathy.

When analyzing the surgical indications, we found that intraoperative retinal tears were less common when the indication for surgery was ERM than for other indications (*p* = 0.043, Table 5). Furthermore, multivariate analysis of variance (MANOVA) did not show any statistically significant association between the DR type or surgery indication with intraoperative or postoperative complications (Table 6).

**Table 5:** Intra- and postoperative complications of combined phaco-vitrectomy in diabetic patients in regard to indication for surgery.

	**VH** **(*N* = 46)**	**ERM** **(*N* = 27)**	**TRD** **(*N* = 15)**	**RRD** **(*N* = 6)**	**VMT** **(*N* = 4)**	***P*-value**
**Any intraoperative complication**	8 (17)	1 (4)	6 (40)	2 (33)	1 (25)	**0.025**
**Any postoperative complication**	29 (63)	17 (63)	12 (80)	4 (67)	2 (50)	0.716
Posterior capsule tear	2 (4)	1 (4)	1 (7)	–	–	1.000
Retinal tear	6 (13)	1 (4)	5 (33)	2 (33)	1 (25)	**0.043**
Corneal edema	3 (7)	1 (4)	–	–	–	0.491
Postoperative macular edema	9 (20)	10 (37)	4 (27)	3 (50)	1 (25)	0.327
Epiretinal membrane	9 (20)	3 (11)	2 (13)	3 (50)	1 (25)	0.258
Neovascular glaucoma	3 (7)	1 (4)	–	–	–	0.491
Persistent inflammation	2 (4)	1 (4)	–	–	–	0.744
Persistent ocular hypertension	8 (17)	2 (7)	2 (13)	–	–	0.720
Posterior capsule opacification	3 (7)	2 (7)	3 (20)	1 (17)	–	0.389
Posterior synechiae	2 (4)	1 (4)	2 (13)	–	–	0.696
Retinal detachment	3 (7)	1 (4)	2 (13)	1 (17)	–	0.578
Vitreous hemorrhage	6 (13)	2 (7)	1 (7)	–	–	0.900

Data is given as absolute numbers with proportions. For multiple group comparisons, categorical data was analyzed with the Fisher-Freeman-Halton test. VH, vitreous hemorrhage; ERM, epiretinal membrane; TRD, tractional retinal detachment; RRD, rhegmatogenous retinal detachment; VMT, vitreomacular traction. *p*-values < 0.05 were considered statistically significant.

**Table 6:** Multivariate analysis of variance (MANOVA) of combined phaco-vitrectomy in patients with NPDR and PDR.

	**Any intraoperative complication**	**Any postoperative complication**
DR type (NPDR/PDR)	−0.361 to 0.076; *P* = 0.199	−0.200 to 0.170; *P* = 0.876
Age (years)	−0.713 to 9.455; *P* = 0.091	−0.677 to 7.892; *P* = 0.098
Indication for surgery	−1.189 to 0.482; *P* = 0.403	−0.981 to 0.423; *P* = 0.432

Data is given as 95% CI and *P*-value. For two-group comparisons, categorical data was analyzed with the Pearson's chi-square test or the Fisher's exact test when values in any of the cells of a contingency table were below five. Indication for surgery we as follows: vitreous hemorrhage (*N* = 46), epiretinal membrane (*N* = 27), tractional retinal detachment (*N* = 15), rhegmatogenous retinal detachment (*N* = 6), and vitreomacular traction (*N* = 4). NPDR, non-proliferative diabetic retinopathy; PDR, proliferative diabetic retinopathy.

## Discussion

Diabetic patients are at higher risk for phacovitrectomy complications, when compared to patients without diabetes (9, 25). Our study investigated visual outcomes and intra- and postoperative complications in phacovitrectomy procedures in diabetic patients. We found high rates of postoperative maculae edema in patients with either NPDR or PDR.

In our study, most patients referred for combined surgery suffered from PDR. This can be attributed to the progressed level of the disease, which entails a higher risk for developing complications calling for surgical intervention. Both groups exhibited a relatively low rate of major complications other than macular edema, which is attributable, in our opinion, to new advanced surgical techniques, and appropriate measures prior to and during the operation. Importantly, none of our patients suffered severe adverse events such as uncontrollable hemorrhage, choroidal effusion or endophthalmitis.

The most common intraoperative complication in both the NPDR and PDR groups was iatrogenic retinal tear. Several other studies investigating phacovitrectomy in various vitreoretinal diseases, including DR, reported lower rates between 5.1 and 5.3% of iatrogenic retinal tear (23, 25), but rates as high as 19% were also reported (24). As expected, in our study retinal tears were less common when the indication for surgery was ERM, as these surgeries are usually more elective and shorter in duration, whereas surgeries in PDR patients included cases of TRD, VH and worse visualization of the retina, explaining higher rates of intraoperative retinal tears, which were in part iatrogenic, especially during the membrane dissection phase in TRD patients.

In our study, the rate of posterior capsular tear was 5% in the PDR group, which was higher than the 2.1% reported by Wensheng et al. (14). As the percentage of eyes with VH in our study population was higher, we hypothesized that the lack of retroillumination during the cataract surgery could raise the rate of posterior capsular tears in the first step of the combined procedure. However, as represented in the study, VH as the indication for surgery was not associated with higher rates of retinal tears when compared to other indications for phacovitrectomy surgery. Previous studies have indicated that PDR eyes have higher risk of developing inflammatory reactions in the anterior chamber (AC) (21, 24, 26–29). In this study, relatively low rates of persistent inflammatory reaction in the AC (3%) or posterior synechiae (5%) were reported when compared to other studies reporting rates between 7.9 and 30.8% (9, 15, 21, 22).

The most common postoperative complication in our study was macular edema, which was diagnosed by OCT in 29% of the NPDR eyes and 26% of the PDR eyes. Our numbers represent higher rates than those presented in other studies, where postoperative macular edema was found in 17.4–19% of eyes in the diabetic population (30, 31). However, Chae et al. demonstrated that clinically meaningful postoperative ME developed in 33.3% of cataract surgery patients with NPDR, who did not have significant macular edema preoperatively (18). In a recent large multicenter retrospective study, Chu et al. represented that postoperative ME incidences can be up to 7.27% in patients with any DR, and up to 12.07% in patients with PDR (11). This particular study was retrospective and did not include patients with prophylactic NSAIDs. Nevertheless, it seems that both diabetes, combined operation (phacovitrectomy) and other retinal comorbidities act as risk factors for postoperative ME. Furthermore, it seems that anti-inflammatory medication with topical steroid or NSAID drops, or their combination is not sufficient to counteract postoperative macular edema in this patient population (32, 33). In our study, about a third of patients received adjunct anti-VEGF injections at the end of the surgery and about 60% of patients received additional panretinal photocoagulation during the surgery, which can exacerbate postoperative macular edema. Several small studies showed that diabetic patients receiving anti-VEGF injections at the end of surgery had less postoperative macular thickening than those who did not (12, 18). In a recently published meta-analysis, Zhang et al. showed that intraoperative injection of anti-VEGF was associated with better BCVA outcomes at 1 month and 3 months postoperatively, but not at 6 months after surgery (34). The ESCRS PREMED Study Report 2 showed that intravitreal triamcinolone, but not bevacizumab, had a significant effect in preventing macular thickness (35). Unfortunately, it is beyond the scope of our study to evaluate the effect of anti-VEGF injections and PRP on postoperative macular edema due to a potential selection bias. All our patients received topical NSAIDs for 3 months postoperatively, yet more than a quarter of the eyes developed postoperative ME. High rates of new-onset macular edema after phacovitrectomy in diabetic patients might warrant further studies to examine the effect of different interventions during the surgery including triamcinolone or anti-VEGF, and perhaps topical NSAIDs after the surgery are not sufficient to prevent postoperative macular edema. Of note, as all our patients underwent OCT imaging, the high ME rate may include clinically non-significant cases as well. Finally, we believe it is important to emphasize that postoperative ME is mostly self-limiting and in a minority of cases with long-term refractory CME, we recommend intravitreal administration of triamcinolone (36).

Neovascular glaucoma was previously described as a complication of phacovitrectomy in diabetic patients (15). In our study, 3 out of the 104 patients (2.9%) developed NVG postoperatively, all in the PDR group. Our results are comparable with the current literature, where postoperative NVG ranges between 0 and 5.3% (15, 16, 25). We attribute the relatively low rate of postoperative NVG in our study to aggressive PRP prior to and during the surgery. Preoperatively, BCVA in the PDR group was significantly lower than in the NPDR group, as expected. Nevertheless, at all postoperative timepoints BCVA was comparable between the NPDR and PDR groups. One explanation for this is the detrimental impact of VH on the patients' visual acuity and its resolution postoperatively, as more than half of the patients in the PDR group were referred to surgery due to VH indication.

Our study has some limitations. First, the patients' medical files were examined for outcomes and complications for 6 months postoperatively. Although most postoperative complications would have probably surfaced during this follow-up period, other DR and surgery-related complications might have emerged after a longer follow-up. Second, preoperative cataracts were not graded, and refractive outcomes of the patients were not measured, due to the retrospective nature of this study. Furthermore, during the analysis, we did not separate between the tears in the peripheral retina caused during shaving, or those occurring in the posterior retina during the release of traction and removal of diabetic membrane. As these tears are of different nature, different complications can emerge, thus effecting the outcomes. Moreover, compliance to postoperative medication was not recorded owing to the retrospective nature of the study, and this could have affected the results. In addition, we were unable to exclude the presence of preoperative DME in patients where visualization was poor due to VH, which may have caused overestimation of the postoperative macular edema. Clear differentiation of PCME and DME also presents a challenge in diabetic patients after surgery and may contribute to overestimation of PCME. Thus, to overcome this limitation, we refer to postoperative ME, which is more accurate in this situation to cover both disease entities. The fact that more than a quarter of patients developed macular edema despite topical NSAIDS after surgery is highly suggestive that the edema is more likely secondary to diabetes rather than a post-operative inflammatory drive. The study groups were not balanced by the number of cases due to higher rates of referral of PDR patients to surgery. Finally, due to the retrospective nature of this study, different surgeons used different methods intraoperatively, including the usage of PRP and anti-VEGF according to their personal preference. Due to the limited number of study participants, larger studies are warranted to further support the findings of this study.

In conclusion, we found that combined phacovitrectomy in both NPDR and PDR patients resolved without substantial difference in outcomes between the groups. Nevertheless, high rates of postoperative macular edema were reported in both groups. In the future, randomized clinical trials on phacovitrectomy should focus on the optimization of anti-inflammatory treatment and postoperative follow-up for the prevention of postoperative macular edema.

## Data availability statement

The raw data supporting the conclusions of this article will be made available by the authors, without undue reservation.

## Ethics statement

The studies involving human participants were reviewed and approved by Rabin Medical Center, Israel. Written informed consent for participation was not required for this study in accordance with the national legislation and the institutional requirements.

## Author contributions

All authors listed have made a substantial, direct, and intellectual contribution to the work and approved it for publication.

## Conflict of interest

The authors declare that the research was conducted in the absence of any commercial or financial relationships that could be construed as a potential conflict of interest.

## Publisher's note

All claims expressed in this article are solely those of the authors and do not necessarily represent those of their affiliated organizations, or those of the publisher, the editors and the reviewers. Any product that may be evaluated in this article, or claim that may be made by its manufacturer, is not guaranteed or endorsed by the publisher.

## References

[1] Yau JWY Rogers SL Kawasaki R Lamoureux EL Kowalski JW Bek T. Global prevalence and major risk factors of diabetic retinopathy. Diabetes Care. (2012) 35:556–64. 10.2337/dc11-1909 22301125 PMC3322721

[2] Hamill EB Ali SF Weng CY. An update in the management of proliferative diabetic retinopathy. Int Ophthalmol Clin. (2016) 56:209–25. 10.1097/IIO.0000000000000136 27575769

[3] ETDRS Research Group. Photocoagulation for diabetic macular edema. Early Treatment Diabetic Retinopathy Study report number 1. Early Treatment Diabetic Retinopathy Study research group. Arch Ophthalmol. (1985) 103:1796–806. 10.1001/archopht.1985.01050120030015 2866759

[4] Helbig H. Surgery for diabetic retinopathy. Ophthalmologica. (2007) 221:103–11. 10.1159/000098255 17380064

[5] Klein BEK Klein R Moss SE. Incidence of cataract surgery in the Wisconsin epidemiologic study of diabetic retinopathy. Am J Ophthalmol. (1995) 119:295–300. 10.1016/S0002-9394(14)71170-5 7872389

[6] Blankenship GW Machemer R. Long-term diabetic vitrectomy results: report of 10 year follow-up. Ophthalmology. (1985) 92:503–6. 10.1016/S0161-6420(85)34015-0 2582329

[7] Braunstein RE Airiani S. Cataract surgery results after pars plana vitrectomy. Curr Opin Ophthalmol. (2003) 14:150–4. 10.1097/00055735-200306000-00007 12777934

[8] Biró Z Kovacs B. Results of cataract surgery in previously vitrectomized eyes. J Cataract Refract Surg. (2002) 28:1003–6. 10.1016/S0886-3350(02)01237-3 12036644

[9] Park SP Ahn JK Lee GH. Morphologic changes in the anterior segment after phacovitrectomy for proliferative diabetic retinopathy. J Cataract Refract Surg. (2009) 35:868–73. 10.1016/j.jcrs.2008.12.032 19393886

[10] Silva PS Diala PA Hamam RN Arrigg PG Shah ST Murtha TL. Visual outcomes from pars plana vitrectomy versus combined pars plana vitrectomy, phacoemulsification, and intraocular lens implantation in patients with diabetes. Retina. (2014) 34:1960–8. 10.1097/IAE.0000000000000171 24830822

[11] Chu CJ Johnston RL Buscombe C Sallam AB Mohamed Q Yang YC. Risk factors and incidence of macular edema after cataract surgery: a database study of 81984 eyes. Ophthalmology. (2016) 123:316–23. 10.1016/j.ophtha.2015.10.001 26681390

[12] Zhao LQ Cheng JW. A systematic review and meta-analysis of clinical outcomes of intravitreal anti-VEGF agent treatment immediately after cataract surgery for patients with diabetic retinopathy. J Ophthalmol. (2019) 2019:2648267. 10.1155/2019/2648267 31143469 PMC6501156

[13] Early photocoagulation for diabetic retinopathy. ETDRS report number 9. Early Treatment Diabetic Retinopathy Study Research Group. Ophthalmology. (1991) 98:766–85. 10.1016/S0161-6420(13)38011-7 2062512

[14] Wensheng L Wu R Wang X Xu M Sun G Sun C. Clinical complications of combined phacoemulsification and vitrectomy for eyes with coexisting cataract and vitreoretinal diseases. Eur J Ophthalmol. (2009) 19:37–45. 10.1177/112067210901900106 19123147

[15] Scharwey K Pavlovic S Jacobi KW. Combined clear corneal phacoemulsification, vitreoretinal surgery, and intraocular lens implantation. J Cataract Refract Surg. (1999) 25:693–8. 10.1016/S0886-3350(99)00022-X 10330647

[16] Tseng HY Wu WC Hsu SY. Comparison of vitrectomy alone and combined vitrectomy, phacoemulsification and intraocular lens implantation for proliferative diabetic retinopathy. Kaohsiung J Med Sci. (2007) 23:339–43. 10.1016/S1607-551X(09)70419-X 17606428 PMC11918075

[17] Wu L Fernandez-Loaiza P Sauma J Hernandez-Bogantes E Masis M. Classification of diabetic retinopathy and diabetic macular edema. World J Diabetes. (2013) 4:290. 10.4239/wjd.v4.i6.290 24379919 PMC3874488

[18] Chae JB Joe SG Yang SJ Lee JY Sung KR Kim JY. Effect of combined cataract surgery and ranibizumab injection in postoperative macular edema in nonproliferative diabetic retinopathy. Retina. (2014) 34:149–56. 10.1097/IAE.0b013e3182979b9e 23807186

[19] Royle P Mistry H Auguste P Shyangdan D Freeman K Lois N. Pan-retinal photocoagulation and other forms of laser treatment and drug therapies for non-proliferative diabetic retinopathy: systematic review and economic evaluation. Health Technol Assess. (2015) 19:1–248. 10.3310/hta19510 26173799 PMC4780877

[20] Lövestam-Adrian M Agardh CD Torffvit O Agardh E. Type 1 diabetes patients with severe non-proliferative retinopathy may benefit from panretinal photocoagulation. Acta Ophthalmol Scand. (2003) 81:221–5. 10.1034/j.1600-0420.2003.00050.x 12780397

[21] Shinoda K O'hira A Ishida S Hoshide M Ogawa LS Ozawa Y. Posterior synechia of the iris after combined pars plana vitrectomy, phacoemulsification, and intraocular lens implantation. Jpn J Ophthalmol. (2001) 45:276–80. 10.1016/S0021-5155(01)00319-7 11369378

[22] Pollack A Landa G Kleinman G Katz H Hauzer D Bukelman A. Results of combined surgery by phacoemulsification and vitrectomy. Isr Med Assoc J. (2004) 6:143–6. 15055268

[23] Li W Sun G Wu R Wang X Xu M Sun C. Longterm results after phacovitrectomy and foldable intraocular lens implantation. Acta Ophthalmol. (2009) 87:896–900. 10.1111/j.1755-3768.2008.01383.x 18937822

[24] Diolaiuti S Senn P Schmid MK Job O Maloca P Schipper I. Combined pars plana vitrectomy and phacoemulsification with intraocular lens implantation in severe proliferative diabetic retinopathy. Ophthalmic Surg Lasers Imaging. (2006) 37:468–74. 10.3928/15428877-20061101-04 17152540

[25] Lee DY Jeong HS Sohn HJ Nam DH. Combined 23-gauge sutureless vitrectomy and clear corneal phacoemulsification in patients with proliferative diabetic retinopathy. Retina. (2011) 31:1753–8. 10.1097/IAE.0b013e31820d4057 21555968

[26] Lahey JM Francis RR Kearney JJ. Combining phacoemulsification with pars plana vitrectomy in patients with proliferative diabetic retinopathy: a series of 223 cases. Ophthalmology. (2003) 110:1335–9. 10.1016/S0161-6420(03)00454-8 12867387

[27] Treumer F Bunse A Rudolf M Roider J. Pars plana vitrectomy, phacoemulsification and intraocular lens implantation. Comparison of clinical complications in a combined versus two-step surgical approach. Graefes Arch Clin Exp Ophthalmol. (2006) 244:808–15. 10.1007/s00417-005-0146-9 16328429

[28] Chung TY Chung H Lee JH. Combined surgery and sequential surgery comprising phacoemulsification, pars plana vitrectomy, and intraocular lens implantation: comparison of clinical outcomes. J Cataract Refract Surg. (2002) 28:2001–5. 10.1016/S0886-3350(02)01354-8 12457677

[29] Demetriades AM Gottsch JD Thomsen R Azab A Stark WJ Campochiaro PA. Combined phacoemulsification, intraocular lens implantation, and vitrectomy for eyes with coexisting cataract and vitreoretinal pathology. Am J Ophthalmol. (2003) 135:291–6. 10.1016/S0002-9394(02)01972-4 12614744

[30] Ahmadabadi HF Mohammadi M Beheshtnejad H Mirshahi A. Effect of intravitreal triamcinolone acetonide injection on central macular thickness in diabetic patients having phacoemulsification. J Cataract Refract Surg. (2010) 36:917–22. 10.1016/j.jcrs.2009.12.030 20494761

[31] Kim SY Yang J Lee YC Park YH. Effect of a single intraoperative sub-Tenon injection of triamcinolone acetonide on the progression of diabetic retinopathy and visual outcomes after cataract surgery. J Cataract Refract Surg. (2008) 34:823–6. 10.1016/j.jcrs.2008.01.018 18471640

[32] Lim BX Lim CHL Lim DK Evans JR Bunce C Wormald R. Prophylactic non-steroidal anti-inflammatory drugs for the prevention of macular oedema after cataract surgery. Cochrane Database Syst Rev. (2016) 1: CD006683. 10.1002/14651858.CD006683.pub3 27801522 PMC6464900

[33] Kim SJ Schoenberger SD Thorne JE Ehlers JP Yeh S Bakri SJ. Topical nonsteroidal anti-inflammatory drugs and cataract surgery: a report by the american academy of ophthalmology. Ophthalmology. (2015) 122:2159–68. 10.1016/j.ophtha.2015.05.014 26123091

[34] Zhang R Dong L Yang Q Liu Y Li H Zhou W. Prophylactic interventions for preventing macular edema after cataract surgery in patients with diabetes: a Bayesian network meta-analysis of randomized controlled trials. EClinicalMedicine. (2022) 49:101463. 10.1016/j.eclinm.2022.101463 35747191 PMC9124709

[35] Wielders LHP Schouten JSAG Winkens B van den Biggelaar FJHM Veldhuizen CA Murta JCN., et al. Randomized controlled European multicenter trial on the prevention of cystoid macular edema after cataract surgery in diabetics: ESCRS PREMED Study Report 2. J Cataract Refract Surg. (2018) 44:836–847. 10.1016/j.jcrs.2018.05.015 30055692

[36] Aaronson A Achiron A Tuuminen R. Clinical course of pseudophakic cystoid macular edema treated with nepafenac. J Clin Med. (2020) 9:3034. 10.3390/jcm9093034 32967137 PMC7563612

